# Elucidating host cell response pathways and repurposing therapeutics for SARS-CoV-2 and other coronaviruses

**DOI:** 10.1038/s41598-022-21984-w

**Published:** 2022-11-05

**Authors:** Zhewei Shen, Anna Halberg, Jia Yi Fong, Jingyu Guo, Gavin Song, Brent Louie, Gregory R. Luedtke, Viwat Visuthikraisee, Andrew A. Protter, Xiaoying Koh, Taegon Baik, Pek Yee Lum

**Affiliations:** 1 Auransa Inc., 1726 Edgewood Drive, Palo Alto, CA 94303 USA; 2 Experimental Drug Development Centre, 10 Biopolis Road, #05-01 Chromos, Singapore, 138670 Singapore; 3 Arum Therapeutics Inc., #301, 38 Magokjungang 8-ro 1-gil, Gangseo-gu, Seoul, 07793 South Korea

**Keywords:** Genomics, Gene expression, Transcriptomics

## Abstract

COVID-19, first reported in late 2019, is an ongoing pandemic that has been causing devastation across the globe. Although there are multiple vaccines that can prevent severe symptoms, effective COVID-19 therapeutics are still of importance. Using our proprietary in silico engine, we screened more than 22,000 unique compounds represented by over half a million gene expression profiles to uncover compounds that can be repurposed for SARS-CoV-2 and other coronaviruses in a timely and cost-efficient manner. We then tested 13 compounds in vitro and found three with potency against SARS-CoV-2 with reasonable cytotoxicity. Bortezomib and homoharringtonine are some of the most promising hits with IC_50_ of 1.39 μM and 0.16 μM, respectively for SARS-CoV-2. Tanespimycin and homoharringtonine were effective against the common cold coronaviruses. In-depth analysis highlighted proteasome, ribosome, and heat shock pathways as key targets in modulating host responses during viral infection. Further studies of these pathways and compounds have provided novel and impactful insights into SARS-CoV-2 biology and host responses that could be further leveraged for COVID-19 therapeutics development.

## Introduction

In December 2019, a new coronavirus emerged and rapidly spread around the world, causing a global pandemic on an unprecedented scale. SARS-CoV-2, the virus that causes COVID-19, is a positive-sense single-stranded RNA coronavirus^1^. Due to the virus’s rapid transmission rate, asymptomatic infection, and the emergence of multiple infectious variants^2,3^, the timely development of effective therapeutic modalities has been a global challenge^4^. While efforts in vaccine development have resulted in emergency use authorization of multiple effective vaccines^5^ and the recent approvals of coronavirus targeting anti-viral compounds^6^, we believe that understanding the biology of infection and the host cell responses will allow us to develop novel therapeutics that could be used concomitantly with current antivirals and vaccines to prevent severe diseases. In addition, we also studied other common cold coronaviruses towards the goal of developing pan-coronavirus therapeutics that could slow or stop viral production in host cells^7^.

We used gene expression profiles of both compound and genetic perturbations as probes for understanding the biology of viral replication. Using this approach, we predicted for compounds that may impede viral production in the host cells. Given SARS-CoV-2 is closely related to SARS-CoV-1 and are both from the beta coronavirus family^8,9^ and the abundance of SARS-CoV-1 data, we used gene expression from viral studies of both viruses for our predictions. The approach starts with our proprietary computational engine that analyzes gene expression data of disease cells and chemical and genetic perturbations to generate in silico predictions for possible treatments. Towards this goal, we screened in silico more than 22,000 existing compounds represented by over half a million gene expression profiles, rapidly producing candidate compounds that can be further tested. In summary, we found that chemically perturbing translation initiation or regulation, heat shock, and proteasomal pathways, were effective in controlling viral reproduction in a pan-virus or virus-specific manner. Small molecules like bortezomib, homoharringtonine and tanespimycin that affected these pathways were able to interfere with viral production against SARS-CoV-2 or the common cold viruses HCoV-229E and HCoV-OC43.

## Results

### Gene expression based in silico “transcriptotypic screen” approach

Because of the genomic similarity between SARS-CoV-1 and SARS-CoV-2, we used gene expression profiles of human lung cell lines and peripheral blood from patients infected with either SARS-CoV-1 or SARS-CoV-2 for the predictions. Host cell response profiles from each condition were used independently for the prediction and tested against a library of gene expression profiles of cell responses to over 22,000 unique compounds or gene perturbations individually.

From the initial raw predictions, we executed our first-round selection by ranking the compounds based on the number of times a compound passes the Fisher exact test in each input data independently. We believe this stringent criteria allows us to identify compounds with the strongest potential to affect viral production. We identified a total of 1416 such ranked compounds from over 6,700 potential compounds that can be sourced and therefore tested quickly. In this round of testing, priority was given to groups of compounds representing the same classes of mechanisms of action (MOA) if multiple of which are top ranked. We then added an additional filter to exclusively focus on launched or clinical phase compounds and tested a representative candidate from each MOA of interest. A total of five compounds, namely trametinib (MEK inhibitor), bortezomib (proteasomal inhibitor), homoharringtonine (protein synthesis inhibitor), dasatinib (Bcr-Abl kinase inhibitor), and lacidipine (calcium channel blocker) were selected for testing in SARS-CoV-2 infected Vero cells. Selective index (SI) was used as a measurement of a compound’s therapeutic benefit over risk.

**Figure 1:**
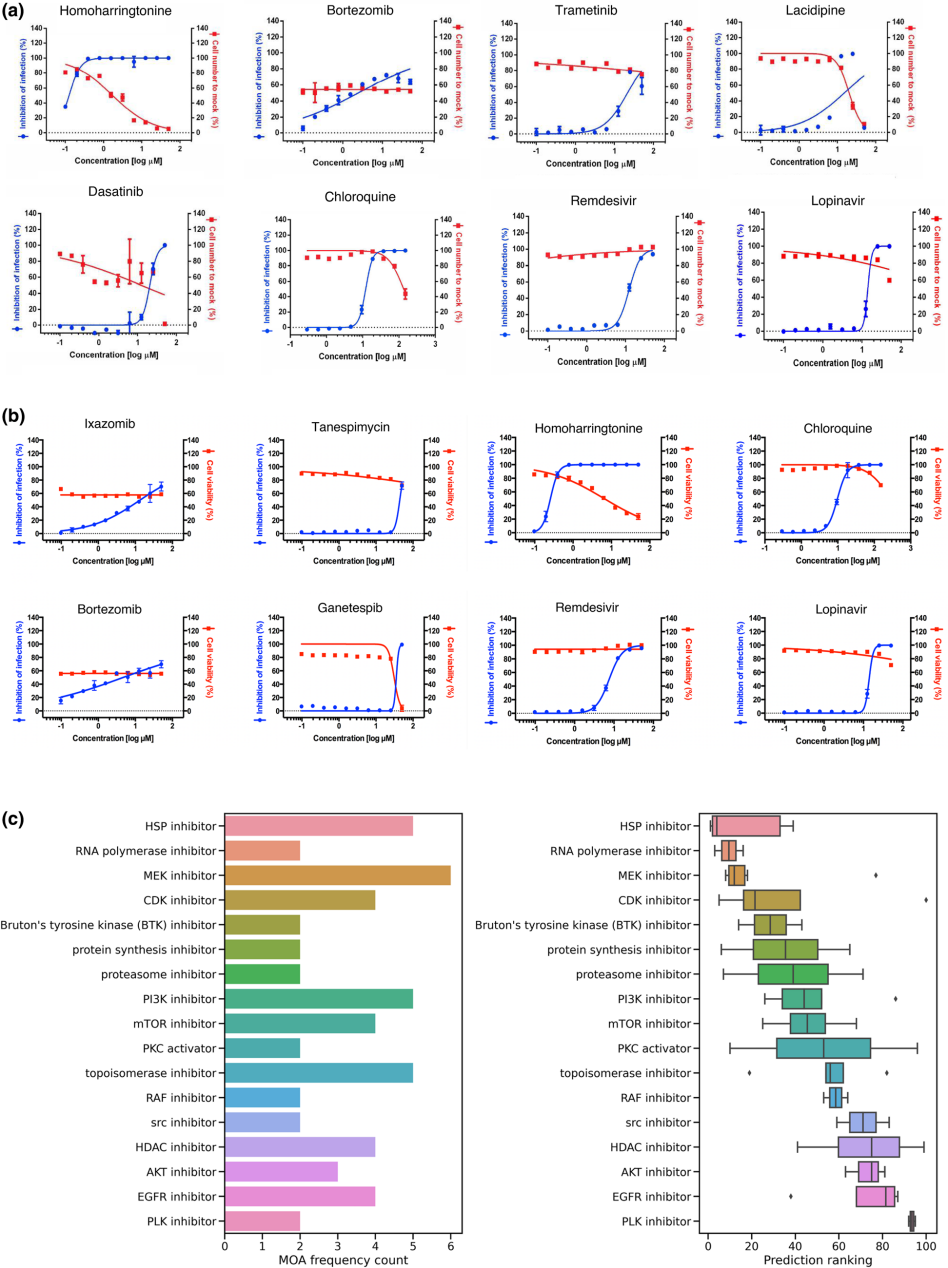
(**A**) Viral inhibition and cell viability curves used to generate Table 1 Round 1. (**B**) Viral inhibition and cell viability curves used to generate Table 1 Round 2. (**C**) Comparison of drug MOA class frequency count and predicted compound prediction ranking in Top 100 compounds with strongest predicted anti-viral strength.

Of the aforementioned compounds, trametinib (SI 3.34) and bortezomib (SI 35.9 and SI 14.63) showed anti-SARS-CoV-2 activities and cytotoxicity over 50 μM (Table 1, Fig. 1a and b). Homoharringtonine is highly effective against SARS-CoV-2 but also exhibits some level of toxicity. However, the SIs of homoharringtonine (12.84 and 24.06, Table 1, Fig. 1a and b) suggest that there may be a reasonable therapeutic window for anti-SARS-CoV-2 activity before cytotoxicity is induced.

**Table 1:** Predicted anti-SARS-CoV-2 compounds in vitro efficacy testing from two independent rounds of testing.

	Compound	IC_50_ (μM)	CC_50_ (μM)	SI index
Round 1	Homoharringtonine	0.16	2.14	12.84
	Bortezomib	1.39	> 50	35.93
	Trametinib	14.95	> 50	3.34
	Lacidipine	14.89	20.15	1.35
	Dasatinib	19.74	13.20	0.67
	Remdesivir	12.08	> 50	4.14
	Chloroquine	12.0	127.8	10.65
Round 2	Ganetespib	35.50	28.95	0.82
	Ixazomib	11.71	> 50	4.27
	Tanespimycin	31.33	> 50	1.60
	Bortezomib	3.42	> 50	14.63
	Homoharringtonine	0.26	6.22	24.06
	Chloroquine	9.80	> 150	15.31
	Remdesivir	7.45	> 50	6.71
	Lopinavir	13.50	> 50	3.70

IC_50_, CC_50_, and SI index were calculated of each compound in SARS-CoV-2 infected in Vero cells. Homoharringtonine and bortezomib exhibited strong antiviral activities with corresponding cytotoxicity CC_50_ at least 20 times higher than the viral killing IC_50_.

The next set of compounds tested was comprised of top ranked compounds that perturb the same pathways. We tested two strongly predicted proteasomal inhibitors namely bortezomib, that showed anti-SARS-CoV-2 activity in the previous experiment, and its analog ixazomib. We also selected two highly ranked compounds from the heat shock pathway, namely tanespimycin and ganetespib. Both the proteasomal pathway and the heat shock pathway were significantly represented among the top 100 ranked compounds (Fig. 1c). Consistent with the initial study, bortezomib and ixazomib showed anti-SARS-CoV-2 activity with a SI of 14.63 and 4.27 respectively (Table 1). Tanespimycin and ganetespib, on the other hand, showed no activity against SARS-CoV-2 in a Vero cell assay despite having very strong prediction scores from the engine (Table 1, Fig. 1b). The potent anti-viral activity of homoharringtonine was consistent across the two independent studies (Fig. 1a and b).

**Figure 2:**
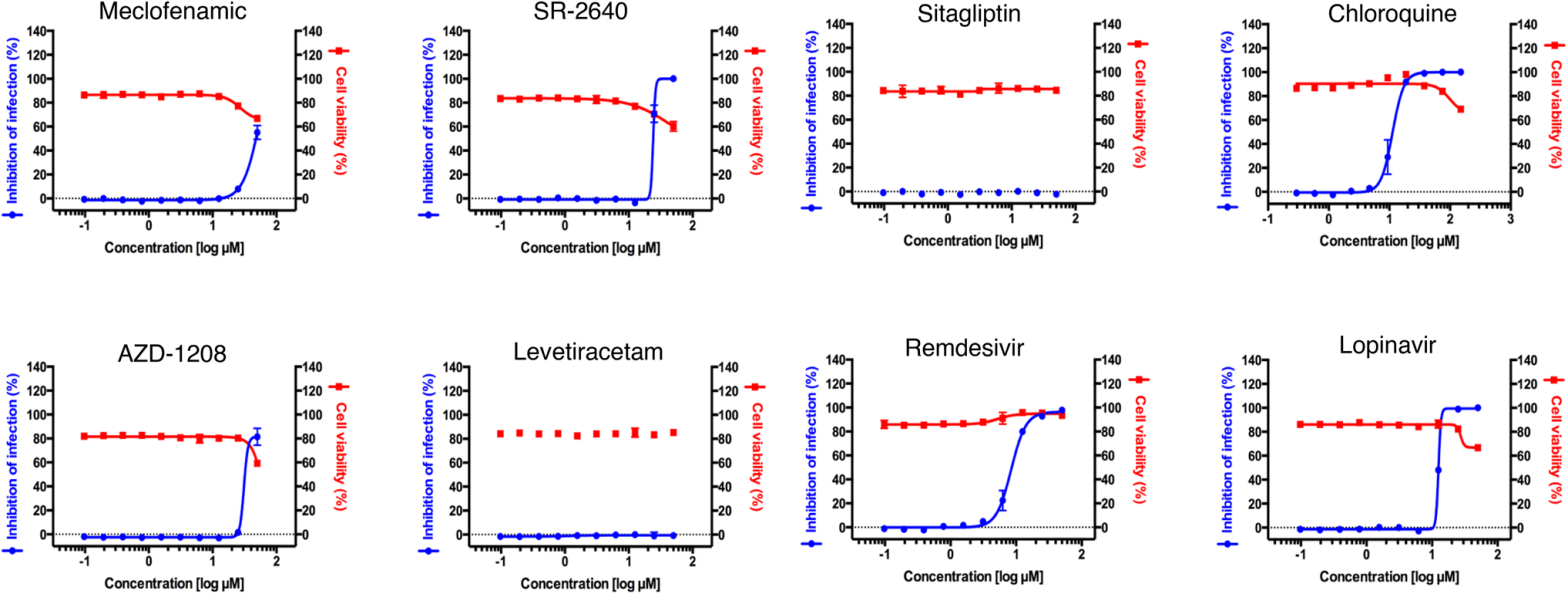
Viral inhibition and cell viability curves used to generate Table 2.

As another test elucidating the biology of host responses to SARS-CoV-2, we identified several compounds that were weakly predicted (poorer ranks) but with novel MOAs. Meclofenamic acid (nonsteroidal anti-inflammatory), sitagliptin (DPP4 antagonist), and levetiracetam (anti-convulsant, MOA unclear) did not show meaningful anti-SARS-CoV-2 activity while SR-2640 (leukotriene D4 and E4 receptor antagonist) and AZD-1208 (PIM kinase inhibitor) had very poor SI values (Table 2, Fig. 2).

**Table 2:** Average reversal score predicted anti-SARS-CoV-2 compounds in vitro efficacy testing.

Compound	IC_50_ (μM)	CC_50_ (μM)	SI index
AZD-1208	27.34	> 50	1.83
Sitagliptin	> 50	> 50	1.00
SR-2640 hydrochloride	24.23	> 50	2.06
Levetiracetam	> 50	> 50	1.00
Meclofenamic acid sodium salt	35.13	> 50	1.42
Chloroquine	11.29	> 150	13.29
Lopinavir	12.63	> 50	3.96
Remdesivir	8.58	> 50	5.83

IC_50_, CC_50_, and SI index were calculated of each compound in SARS-CoV-2 infected in Vero cells. . No compound exhibited reasonable anti-SARS-CoV-2 activities due to high IC_50_ across board.

### Efficacy of compounds against common cold-causing human coronaviruses

**Figure 3:**
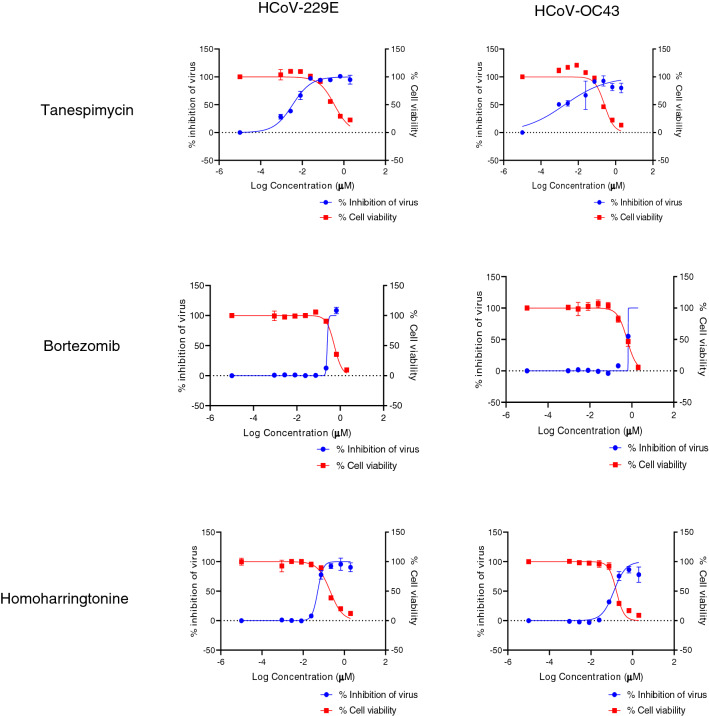
EC_50_ versus CC_50_ curves associated with Table 3 for the 3 compounds tested against HCoV-229E and HCoV-OC43 in MRC5 cells.

We were also interested to understand if these compounds and their target pathways have wider applications in modulating viral host responses, specifically other coronaviruses. The two common cold viruses, HCoV-229E and HCoV-OC43, belong to the coronavirus alpha and beta subgroup, respectively. Both SARS-CoV-1 and SARS-CoV-2 are also in the beta subgroup^8,9^. We tested bortezomib, tanespimycin, and homoharringtonine against these two cold viruses in a human lung fibroblast cell line. Our results show strain specific sensitivity to these compounds. Bortezomib showed no activity against HCoV-229E or HCoV-OC43 in three independent studies (Table 3, Fig. 3), though it has favorable SIs against SARS-CoV-2 (Table 1). Tanespimycin, on the other hand, was highly effective against HCoV-229E and HCoV-OC43 with an EC_50_ of less than 1 nM (Table 3, Fig. 3), though it did not exhibit anti-SARS-CoV-2 activity in Vero cell studies (Table 1). Homoharringtonine was effective against HCoV-229E and HCoV-OC43 with EC_50_s < 100 nM (Table 3, Fig. 3), as well as against SARS-CoV-2 with an average EC_50_/IC_50_ under 300 nM (Table 1).

**Table 3:** (A) EC_50_, CC_50_, and SI index of 3 compounds of interest in MRC5 cells infected with HCoV-229E or HCoV-OC43.

HCoV strain	Compound	EC_50_ (μM)	CC_50_ (μM)	SI index
HCoV-229E	Tanespimycin	0.0036	0.182	101
	Bortezomib	0.2518	0.5364	2
	Homoharringtonine	0.05106	0.2046	4
HCoV-OC43	Tanespimycin	0.0019	0.2528	133
	Bortezomib	0.666	0.5914	< 1
	Homoharringtonine	0.1261	0.1670	1

Tanespimycin and homoharringtonine exhibited anti-HCoV-229E with tanespimycin’s EC_50_ 101 times lower than its CC_50_ and homoharringtonine 4 times lower. Tanespimycin was also effective against anti-HCoV-OC43 with an EC_50_ 133 times lower than its CC_50_. Bortezomib exhibited no anti-viral activity against either strain.

### Pathways predicted that are amenable to small molecule intervention

**Figure 4:**
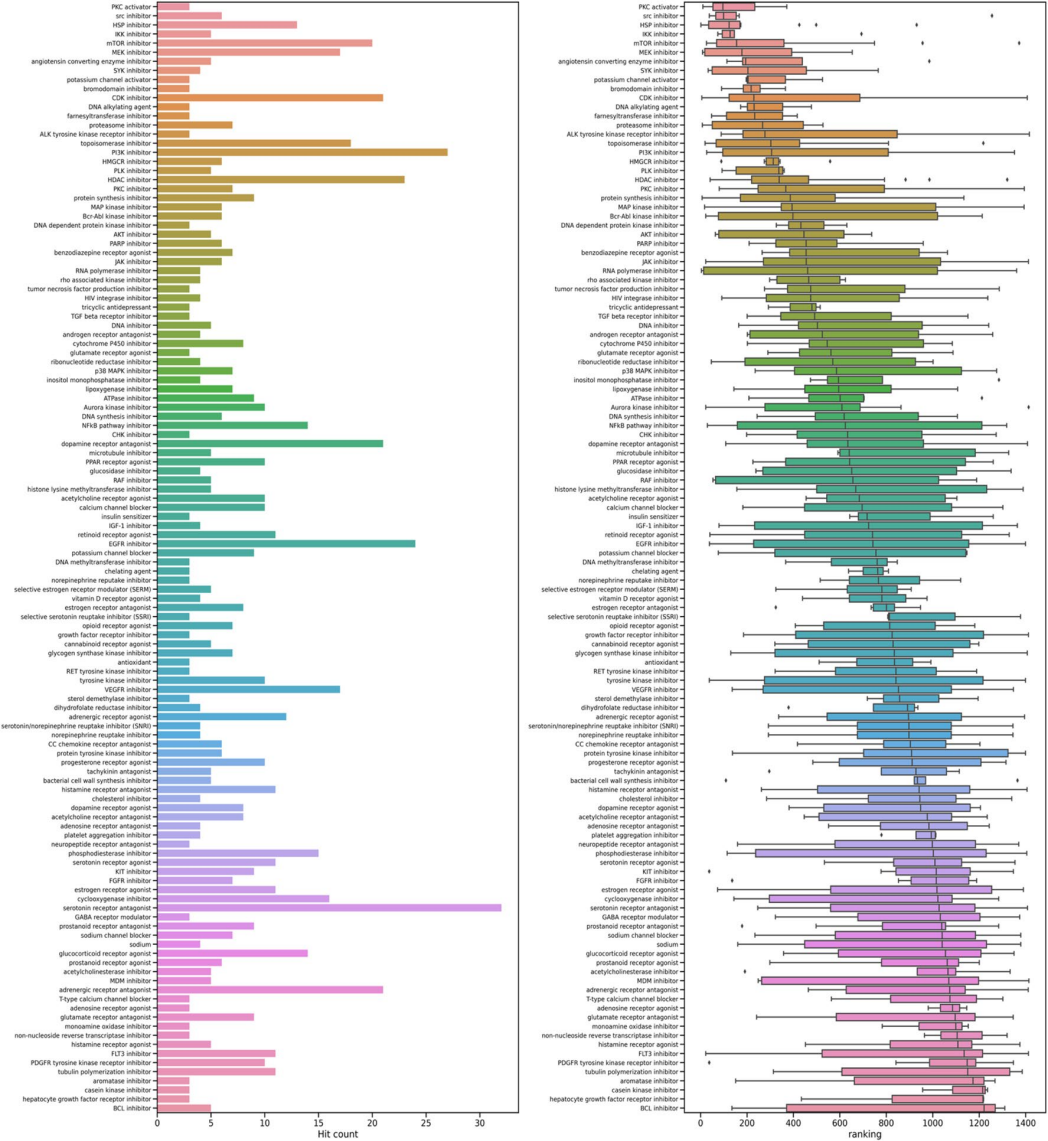
Visualization of drug class frequency count and prediction strength ranking of all predicted anti-SARS-CoV-2 compounds. The predicted drug MOAs summary and ranking are useful tools to generate insights into the biological pathways important to SARS-CoV-2 infected host cells. The top list of most reoccurring MOAs and rankings of predicted compounds consisted of immune type (IKK inhibitor), anti-cancer type (mTOR and MEK inhibitor), and unfolded protein response/UPR type (proteasome inhibitor, HSP inhibitor). We postulate that the anti-cancer and UPR compounds may be linked to the host cell responses to rapid viral replication while the immune type compounds reflect the antiviral inflammatory responses generated by the host cells.

In addition to the compounds we tested, we also investigated the pathways that are represented by the top 100 ranked predicted compounds. Of the top compounds with known MOAs, we found that compounds representing the heat shock, proteasomal, and protein synthesis pathways are ranked very highly in our approach in addition to being effective in curtailing viral production in our experiments (Fig. 1c). In contrast, compounds representing the neurotransmitter related classes (e.g., serotonin receptor antagonists, dopamine receptor antagonists) and inflammatory (lipoxygenase, histamine, chemokine) are often represented by more lowly ranked compounds (Fig. 4). As mentioned earlier, both levetiracetam and SR-2640 hydrochloride did not result in a meaningful effect on viral production (Table 2).

### Using expression profiles of gene knockdowns to probe host response biology

By processing the gene expression profiles of gene knockdowns in the same way as we did for compound-generated gene expression profiles, we generated a list of genes whose knocked- down gene expression profiles were predicted by the Auransa engine to show strong anti-SARS-CoV-2 activities. Pathway analysis using predicted gene targets found a significant enrichment of proteasome pathway and heat shock pathway with p-values of 0.01 and 0.07 from hypergeometric tests, respectively. In fact, shRNA knockdown of HSP90AB1 is ranked the 14th most likely candidate to reverse SARS-CoV-2 signatures in host cells.

### Deconvoluting the biology of the prediction

In order to better understand the MOA underlying the compounds that were predicted and showed efficacy, we explored the biological rationale underlying the prediction that yielded the three viable compounds, focusing on pathways that were deemed important by our engine as it made those predictions (Table 1). Our algorithm found that NF-kappaB signaling, proteasomal/ER stress activities, and cell cycle regulation are key targets affected by bortezomib. Tanespimycin shows in silico effects on heat shock pathways, oxidative stress responses, lipid metabolism, and cell cycle. Homoharringtonine has impacts on a bigger set of biological processes including cholesterol and steroid biosynthesis, cell cycle, and NF-kappaB signaling (Table 4).

**Table 4:** Selective pathways identified using top genes predicted by Auransa engine to be reversed by anti-viral compounds based on STRING DB analysis.

Compounds	Pathway name	Source	Strength	FDR
Bortezomib	Canonical NF-kB pathways, and NF-kappa-B/Dorsal	STRING	1.81	0.00055
	G2/M DNA replication checkpoint	REACTOME	1.99	0.0267
	I-kappaB/NF-kappaB complex	GO-CC	1.99	0.0018
Homoharringtonine	I-kappaB/NF-kappaB complex	GO-CC	1.92	0.0013
	Canonical NF-kB pathways, and NF-kappa-B/Dorsal	STRING	1.81	0.00082
	Negative regulation of reactive oxygen species metabolic process	GO-BP	1.23	0.0105
Tanespimycin	Chaperone-mediated protein transport	GO-BP	1.73	0.0071
	Cholesterol biosynthesis	UniProt	1.47	0.0137
	Steroid biosynthesis	UniProt	1.65	0.00114

A clear NF_k_B signature was likely the driver for predicting bortezomib and homoharringtonine as anti-viral compounds in host cells. In contrast, we hypothesize that tanespimycin targets the metabolism and protein folding/synthesis part of viral biology.

## Discussion

We used our proprietary computational engine to predict compounds and targets in order to study host cell responses to infections by SARS-CoV-1, SARS-CoV-2 and 2 common cold coronaviruses. We showed that the most strongly predicted compounds belong to the classes of compounds known to modulate the following pathways: PKC, SRC, HSP, IKK, mTOR, ACE and proteasome (Fig. 4). Interestingly, the most frequently predicted drug class among the 1416 unique compounds are the neurotransmitter related classes (e.g., serotonin receptor antagonists, dopamine receptor antagonists). However, these compounds generally have lower rankings compared to compounds from the heat shock, proteasomal and gene translation classes (Fig. 4). Levetiracetam, one of the compounds in the most frequent but low-ranking category, had no anti-viral activity (Table 2, Fig. 2). In contrast, the positive results we obtained are from highly ranked predicted compounds belonging to the cell growth, proteasome, and heat shock drug classes. These results suggest that frequency of drug classes may not be as important for compound selection as much as the predicted ranks that are based on the engine’s disease reversal strength scores (Fig. 4). Counting drug class frequency also has the bias of more frequently annotated, perhaps older and larger classes of popular compounds compared to newer or smaller classes of compounds.

Many compounds have been predicted or tested for potential activities against SARS-CoV-2 since the start of the pandemic by other research groups. Numerous early- and late-stage ongoing clinical trials are focused on compounds mostly belonging to the anti-inflammation or direct anti-viral growth classes^10^. Our engine identified some of these reported anti-SARS-CoV-2 compounds, specifically emetine (protein synthesis inhibitor), niclosamide (anthelminthic), and pralatrexate (antineoplastic folate analog metabolic inhibitor)^11–13^. Niclosamide and emetine showed impressive anti-SARS-CoV-2 capabilities in published preclinical studies, with reported SI index of 178.57 and 176.65, respectively. They are ranked #6 and #28 in our predictions. Pralatrexate, with a reported IC_50_ of 0.008 μM^13^, is ranked within the top 60%.

One interesting pathway in our in silico prediction is the proteasome pathway. The proteasome inhibitors bortezomib and ixazomib showed efficacy in controlling viral load without significant cytotoxicity. Of the two, bortezomib exhibited more potency. The ubiquitin–proteasome pathway is a key pathway regulating a variety of cellular processes. Viruses are known to hijack this pathway for their propagation^14^. Interestingly, we did not find bortezomib to be effective against the cold viruses. It is unclear as to why ubiquitin–proteasome pathway inhibition had no effect in our anti-cold viral assays. One reason could be virus specific susceptibility to proteasome inhibition. We also cannot rule out cell type specificity having an impact on the results of the in vitro assay. Furthermore, whether proteasome inhibitors can be developed as an anti-viral depends on their efficacy vs. toxicity profiles in vivo. We took up the exercise of calculating the clinical exposure of bortezomib from publicly known clinical data and found it to be approximately 580 nM via intravenous infusion for cancer (see Materials and Methods). IC_50_s of bortezomib in the in vitro assays are 1.39 μM and 3.42 μM with CC_50_ > 50 μM, indicating that there may be a reasonable window of safety.

Several publications have also mentioned bortezomib as a potential candidate either by different in silico approaches or via compound screening in the laboratory. The in silico approaches ranged from prediction only to analyzing transcription factors regulatory and protein–protein interactions networks. Pan et al*.* and Adhami et al*.* used preselected gene sets representing viral responses to predict anti-COVID-19 compounds^15,16^. Xing et al*.* utilized known antivirals as internal comparisons to identify and test compounds that can significantly reverse COVID-19 viral signature^17^. These different approaches all led to bortezomib as a candidate for anti-SARS-CoV-2. These studies, together with our unsupervised (without a known training set, preselection of genes, or previously tested positive compound ‘hits’) in silico approach, further validate the proteasomal pathway as a critical pathway that can be targeted as an anti-viral strategy.

The next pathway of significance in our prediction is RNA translation. We predicted and validated in vitro that ribosome inhibitor homoharringtonine is highly effective against both SARS-CoV-2 and cold coronaviruses. Schubert et al*.* showed that the Nsp1 (also known as the host shutoff factor) interferes with the ribosomal mRNA channel to stop translation of host mRNAs as a host defense against SARS-CoV-2 infection^18^. Although targeting something as critical as RNA translation may adversely impact the host cells, we believe that homoharringtonine, an inhibitor of the ribosome complex, should be further studied as a potential antiviral drug. Even though stopping RNA translation can be detrimental for both the host cell and virus, SIs of homoharringtonine (12.84 and 24.06, Table 1, Fig. 1a and b) indicate that there might be enough of a window to give the host cell an edge by stopping the very mechanisms that the virus relies on to replicate. Other reports using in vitro cell based screening of FDA approved drugs such for TMPRSS2 reduction or in silico prediction to affect IFN-beta genes also identified homoharringtonine as a promising candidate^19,20^. Similar to bortezomib, these studies, together with the independent identification by our engine, indicates that gene translation is a critical machinery used by the virus to replicate. We also show that homoharringtonine is potent against HCoV-229E and HCoV-OC43 with EC_50_s < 100 nM, indicating perhaps RNA translation may be a common theme that can be exploited as a pan anti-coronavirus strategy. In addition, we calculated the clinical exposure of homoharringtonine to approximately 66 nM. IC_50_s of homoharringtonine is 0.16 μM and 0.26 μM in our testing (see Materials and Methods), hence we also believe that additional effort is warranted to explore homoharringtonine as a potential pan anti-coronavirus compound, although known adverse effects of homoharringtonine must be considered^21^. However, it is also worth noting that a clinical trial in COVID-19 patients treated with nebulized homoharringtonine is ongoing^22^.

The last compound, tanespimycin, was predicted very strongly from the SARS-CoV-1 and SARS-CoV-2 data but surprisingly, neither tanespimycin nor a second generation HSP90 inhibitor ganetespib exhibited anti-viral efficacy in our SARS-CoV-2 assays. In contrast, tanespimycin was highly potent against the 2 cold coronaviruses. Interestingly, Li et al*.* have shown that 10 μM tanespimycin effectively inhibited viral activities of SARS-CoV-1, SARS-CoV-2, and MERS-CoV in Huh-7 cells^23^. We believe that the HSP90 pathway deserves a closer look in other cell types and conditions. Furthermore, we postulate that discrepancy in tanespimycin in vitro efficacy against SARS-CoV-2 may be partly caused by the difference in cell models used (i.e., Vero vs. Huh-7). Prior publication has eluded to anti-SARS-CoV-2 drug response disparity in a cell line dependent manner in Vero and Calu-3 cells^24^.

In 2020, remdesivir, a viral RNA polymerase inhibitor originally developed to treat Ebola patients, was found to show clinical improvement in COVID-19 patients in clinical trials and subsequently approved in the United States in October 2020^25^. We note that remdesivir was not predicted by our approach. This is likely because our approach uses gene expression profiles of infected host cells to predict compounds rather than targeting the viral proteins. Remdesivir, on the other hand, targets the viral RdRp, with many fold specificity over human RNA Polymerase II and mitochondrial RNA polymerase^18–20^, hence, we do not expect to see remdesivir or any other virus-targeting compounds to be predicted using our approach.

Another point worth mentioning is that our in silico predictions are focused on host cell responses to viral infection instead of targeting specific viral proteins, such as the SARS-CoV-2 spike protein. The SARS-CoV-1 and SARS-CoV-2 data used for the predictions consist of genomic signatures of infected host cells, not viral genomic data. We believe that a host-cell based approach allows the predictions to be more versatile and to focus on broader host defense pathways that may be targeted by existing compounds for a faster therapeutic development cycle.

In summary, our approach has pointed us to several critical host cell pathways that could be targeted to stop coronavirus replication, namely the proteasomal pathway, protein synthesis or post-translational regulation machinery, and the heat shock system. We identified compounds in the above pathways that are candidates for repurposing. Although the safety profiles of these potential candidates need to be considered in an anti-viral context, we believe that repurposing drugs with clinical approval or in advanced clinical phases with acceptable clinical safety will greatly shorten the development time and provide the opportune therapies during an ongoing pandemic. In fact, several existing drugs had been recommended to treat hospitalized COVID-19 patients, including antiviral drugs (e.g., remdesivir), anti-inflammatory drugs (e.g., baricitinib and corticosteroids), and intravenous monoclonal antibodies^29^.

We demonstrated that an in silico approach such as ours, done without pre-defined sets of genes or the necessity to train using existing known anti-viral agents, can generate promising anti-viral compounds and vulnerable pathway predictions, with in vitro efficacy and reasonable cytotoxicity. Importantly, our prediction focuses us on compounds and pathways that can modulate host responses in a more pan-coronavirus way instead of inhibiting specific viral proteins. These compounds may have wider clinical applications beyond SARS-CoV-2 treatment as shown in our in vitro results and are less constrained by the virus strains as exemplified by homoharringtonine. Future additional studies of the predicted compounds in human cell lines (e.g., primary human airway epithelium cells or Calu-3) may also provide other insights into differences in efficacy in different biological relevant systems. We believe that our work shows strong support for antiviral therapy development focused on host response regulation.

## Materials and methods

### Data curation and processing

SARS-CoV-1 and SARS-CoV-2 studies used in the analysis were curated from NCBI Gene Expression Omnibus (GEO). The viral infected genomic signatures are included in the Supplementary Table 1.

Gene signatures of tested compounds were curated from NCBI GEO and SRA. The corresponding accession numbers are included in Supplementary Table 2.

RNASeq data were processed from fastq format to TPM values using Salmon^30^. Microarray data were downloaded from NCBI GEO as is but may be further processed according to data processing specifications by the data publishers. If the dataset is published as raw scores, we may apply RMA or quantile transformation as needed. The distribution and scale of all gene expression datasets are then examined to ensure that they are comparable without obvious abnormality and skewness. All datasets prior to compound prediction are confirmed to a log2 scale, which while facilitates log fold change based calculation, also reduces the influence of potential extreme values. Log fold change (LogFC) values were computed by contrasting a total of 14 selected viral infection conditions against corresponding controls.

Auransa’s curated gene expression database of compounds and genetic perturbations comprise of over half a million gene expression profiles across over 22 K unique compounds (drug induced gene expression signature, DIGS). The DIGS data were used to assess compound reversal strength against the gene expression profiles generated from human cells infected with viruses of interest. All publicly available gene expression profiles were downloaded from the GEO.

### Computational compound prediction

For compound prediction, we designed our prediction algorithm to score and select the compounds that maximize the reversal effect of the human genomic expression under virus infection, such that these compounds may have the potential to correct the patient phenotypes under virus infection. This algorithm is, in part, based on the concept of GSEA^31^. All genes in the gene expression profiles were used in the algorithm, without any pre-selection that might bias the results. Our algorithm also does not need to be trained on any pre-existing list of known antiviral compounds, hence opening us up to discovering compounds and critical pathways in an unsupervised manner.

After the reversal scores were calculated, we used a Fisher Exact test-based method for ranking. The Fisher Exact test was used to examine if any single compound has a significant good-hit-bad-hit count ratio compared to all other compounds within the same DIGS. FDR correction was performed and filtered at the significance threshold of 0.05. The threshold of FDR was applied for all compounds tested in silico on each contrast, i.e., a Benjamini/Hochberg FDR was calculated for multiple testing correction. Each time a compound has a significant FDR value, it is considered a hit count of 1. The Fisher Exact test was conducted on each contrast individually where they were treated independently without averaging across studies. Finally, the total number of counts across all input conditions is summed into a single cumulative sum for ranking. To break the ties in ranking, we also calculate the percentage of number of drug signatures fulfilling the reversal score threshold / total number of drug signatures for a single compound for contrasts where the Fisher Exact test yielded a significant result. The 50 top ranked approved compounds are listed in Supplementary Table 3.

We also tested compounds that may not pass the method above but nonetheless exhibited on average a good reversal score across 14 contrasts on the drug signature level and is of novel MOAs. An average reversal score was computed for each drug signature and then filtered using the predetermined reversal score threshold. These drug signature conditions were ranked based on the average score in an ascending manner. We selected a few compounds in this category to represent each pathway of interest.

### Pathway enrichment analysis

Top 100 most significant genes identified by Auransa engine in our approach as described in the earlier section were analyzed using STRING database^32,33^ (STRING DB, https://string-db.org/).

### Compound sourcing

All compounds are research grade chemicals sourced from MedChemExpress, Sigma-Aldrich, Tocris Bioscience, and Selleck Chemicals.

### Anti-SARS-CoV-2 compound testing

Predicted compounds were tested at the Institut Pasteur Korea (IPK, Seongnam, South Korea). The detailed assays employed by IPK are described in Jeon et al.^34^. In short, Vero cells were infected with SARS-CoV-2 for 24 h followed after a 1-h preincubation with the compounds, all of which were done in triplicates. Viral loads were determined by immunofluorescence staining of SARS-CoV-2 Nucleocapsid protein. Fluorescence expression was imaged using Operetta (Perkin Elmer, Waltham, MA) and analyzed using an institutional proprietary Image Mining (IM) software. Dose response curve values were computed using XLFit 4 software (IDBS, Woking, U.K.). All IC_50_ and CC_50_ values were measured in two repeated experiments.

### Anti-HCoV-229E and HCoV-OC43 compound testing

Human lung fibroblast MRC5 cells were cultured in a 96-well plate at 10,000 cells/well in EMEM + 10% FBS overnight. The next day the culture medium was removed from each well and compounds in EMEM + 2% FBS + 0.5% DMSO were added. Each drug was tested on its own 96 well plate. Each plate contained 3 wells of uninfected cells without DMSO, 3 wells of uninfected cells with 0.5% DMSO, 3 wells of infected cells with no DMSO, and 3 wells of infected cells with 0.5% DMSO. Experimental compounds were tested in triplicate at each concentration. Tanespimycin, bortezomib, and homoharringtonine were tested using 2 µM starting concentration, 8-point, threefold serial dilution. After a 1-h pre-incubation with testing compounds, HCoV-229E or HCoV-OC43 viruses at an MOI of 0.01 were added to the culture with compounds for another 1 h. Cell-Titer Glo assay was added after additional incubation time with compounds of interest (HCoV-229E for 96 h and HCoV-OC43 for 120 h). Assay readout was performed using luminescence measurement on a Tecan Spark plate reader.

CC_50_ values were determined by applying nonlinear fit of luminescence readouts from uninfected, compound-treated wells against compound concentration. Likewise, EC_50_ values were determined by nonlinear fit of luminescence readouts from virus-infected, compound-treated wells. Nonlinear regression models were applied for derivation of EC50 and CC50 values respectively, based on built-in equations of Prism Version 8.2.1 (GraphPad Software, San Diego, CA). SI calculated as the ratio of CC50 to EC50.

### Clinical dosing estimation of compounds

**Table:** 

Drug	Anti-Covid IC_50_	Known Pharmacology	Clinical Exposure	References
Lacidipine^1^	14.89 μM	Calcium channel blocker	0.003–0.013 μM	^35^
Trametinib^2^	14.95 μM	MEK1/2 inhibitor	0.045 μM	^36^
Homoharringtonine^3^	0.16 μM	Inhibition of protein synthesis	0.046 μM	^37^
Bortezomib^4^	1.39 μM	Reversible inhibitor of the chymotrypsin-like activity of the 26S proteasome	0.29 μM	^38^

^1^Cmax after a single dose of 4 mg per individual ranged from 1.6 to 5.7 ng/mL; molecular weight is 455.5 ng/nmol^35^

^2^Cmax after a single dose of 2 mg per individual is 27.6 ng/mL; molecular weight is 615 ng/nmol^36^

^3^Cmax after a single dose of 1.25 mg/m2 is: 25.1 ng/mL; molecular weight is 545.6 ng/nmol^37^

^4^Cmax after the first dose of 1.3 mg/m2 is: 112 ng/mL; molecular weight is 384.24 ng/nmol^38^

## Supplementary Information

Supplementary Information.

## Data Availability

SARS-CoV-1 and SARS-CoV-2 genomics data can be found on Gene Expression Omnibus (GEO). The following datasets were used as viral infected genomic profiles: GSE17400, GSE47960, GSE47961, GSE47962, GSE1739, GSE5972, PRJNA625518, PRJNA631969, and PRJNA637580. The following values include drug treatment genomic signatures for compounds predicted and tested: Bortezomib, GSE48056,GSE92742; Chloroquine, GSE116023,GSE92742; Dasatinib, GSE92742,GSE39073; Ganetespib, GSE92742; Homoharringtonine, GSE92742; Ixazomib, GSE66415,GSE66417,GSE92742; Lacidipine, GSE92742; Levetiracetam, GSE92742; Lopinavir, GSE92742; Meclofenamic acid sodium salt, GSE92742; Remdesivir, GSE154936; Sitagliptin, GSE92742; SR-2640 hydrochloride, GSE92742; Tanespimycin, GSE92742; Trametinib, GSE98399,GSE112282,GSE114060,GSE92742. Drug prediction results against all SARS-CoV-1 and SARS-CoV-2 infected cohorts described in this study are hosted on a public facing web application (https://covid19.public.auransa.com/). Users can select compounds of interest to query their Fisher Exact test results in relevant disease cohorts. This web service also provides basic compound annotations for queried compounds using PubChem data^39^.
