# Supplementary material for: Elucidating host cell response pathways and repurposing therapeutics for SARS-CoV-2 and other coronaviruses
Source: Sci Rep. 2022 Nov 5;12:18811. doi: 10.1038/s41598-022-21984-w (PMC9637228; doi:10.1038/s41598-022-21984-w)
Supplement: Supplementary file 1 — Supplementary Information. [file 41598_2022_21984_MOESM1_ESM.docx]

| GSE17400 |
| --- |
| GSE47960 |
| GSE47961 |
| GSE47962 |
| GSE1739 |
| GSE5972 |
| PRJNA625518 |
| PRJNA631969 |
| PRJNA637580 |

**Supplementary Table 1**

**Supplementary Table 1:** Table of GEO accessions and BioProject IDs of viral profiles. The table contains all GEO accessions and BioProject IDs used to generate SARS-CoV-1 and SARS-CoV-2 infected genomic profiles.

## **Supplementary Table 2**

| **Drugs** | **GSE used for gene signatures** |
| --- | --- |
| Bortezomib | GSE48056,GSE92742 |
| Chloroquine | GSE116023,GSE92742 |
| Dasatinib | GSE92742,GSE39073 |
| Ganetespib | GSE92742 |
| Homoharringtonine | GSE92742 |
| Ixazomib | GSE66415,GSE66417,GSE92742 |
| Lacidipine | GSE92742 |
| Levetiracetam | GSE92742 |
| Lopinavir | GSE92742 |
| Meclofenamic acid sodium salt | GSE92742 |
| Remdesivir | GSE154936 |
| Sitagliptin | GSE92742 |
| SR-2640 hydrochloride | GSE92742 |
| Tanespimycin | GSE92742 |
| Trametinib | GSE98399,GSE112282,GSE114060,GSE92742 |

**Supplementary Table 2**: Table of GEO accessions used for compound signatures. GEO accessions used to generate gene signatures of compound treatment for each compound tested for antiviral efficacy.

**Supplementary Table 3**

| **Drugs** | **MOA** |
| --- | --- |
| Ixazomib | proteasome inhibitor |
| Trametinib | MEK inhibitor |
| Dactinomycin | RNA polymerase inhibitor |
| Mitoxantrone | topoisomerase inhibitor |
| Palbociclib | CDK inhibitor |
| Niclosamide | DNA replication inhibitor\|STAT inhibitor |
| Bortezomib | NFkB pathway inhibitor\|proteasome inhibitor |
| Daunorubicin | RNA synthesis inhibitor\|topoisomerase inhibitor |
| Dasatinib | Bcr-Abl kinase inhibitor\|ephrin inhibitor\|KIT inhibitor\|PDGFR tyrosine kinase receptor inhibitor\|src inhibitor\|tyrosine kinase inhibitor |
| Gefitinib | EGFR inhibitor |
| Ibrutinib | Bruton's tyrosine kinase (BTK) inhibitor |
| Gemcitabine | ribonucleotide reductase inhibitor |
| Idarubicin | topoisomerase inhibitor |
| Epirubicin | topoisomerase inhibitor |
| Teniposide | topoisomerase inhibitor |
| Vemurafenib | RAF inhibitor |
| Homoharringtonine | protein synthesis inhibitor |
| Dalfampridine | potassium channel blocker |
| Doxorubicin | topoisomerase inhibitor |
| Vorinostat | HDAC inhibitor |
| Crizotinib | ALK tyrosine kinase receptor inhibitor\|MTH1 inhibitor |
| Atorvastatin | HMGCR inhibitor |
| Tenofovir | HIV integrase inhibitor\|nucleoside reverse transcriptase inhibitor |
| Ingenol | PKC activator |
| Sirolimus | calcineurin inhibitor;mTOR inhibitor |
| Fosfomycin | bacterial cell wall synthesis inhibitor |
| Closantel | chitinase inhibitor\|NFkB pathway inhibitor |
| Olprinone | phosphodiesterase inhibitor |
| Erlotinib | EGFR inhibitor |
| Curcumin | cyclooxygenase inhibitor\|histone acetyltransferase inhibitor\|lipoxygenase inhibitor\|NFkB pathway inhibitor |
| Methylene-Blue | guanylyl cyclase inhibitor\|nitric oxide production inhibitor |
| Panobinostat | HDAC inhibitor |
| Letrozole | aromatase inhibitor |
| Menadione | mitochondrial DNA polymerase inhibitor\|phosphatase inhibitor |
| Telotristat | tryptophan hydroxylase inhibitor |
| Ifosfamide | DNA alkylating agent |
| Pirarubicin | topoisomerase inhibitor |
| Vilazodone | serotonin reuptake inhibitor |
| Loxoprofen | cyclooxygenase inhibitor\|prostanoid receptor antagonist |
| Vismodegib | hedgehog pathway inhibitor\|smoothened receptor antagonist |
| Benazepril | angiotensin converting enzyme inhibitor |
| Gallopamil | L-type calcium channel blocker |
| Delivert | angiotensin receptor agonist |
| Moxifloxacin | bacterial DNA gyrase inhibitor |
| Trifluoperazine | dopamine receptor antagonist |
| Donepezil | acetylcholinesterase inhibitor |
| Zofenopril-Calcium | angiotensin converting enzyme inhibitor |
| Mepacrine | cytokine production inhibitor\|NFkB pathway inhibitor\|TP53 activator |
| Lapatinib | EGFR inhibitor |
| Olaparib | PARP inhibitor |

**Supplementary Table 3:** Table of top 50 predicted anti-SARS-CoV-2 compounds. The 50 top ranked approved compounds and their MOAs are listed.
